# HMGB1, NLRP3, IL-6 and ACE2 levels are elevated in COVID-19 with headache: a window to the infection-related headache mechanism

**DOI:** 10.1186/s10194-021-01306-7

**Published:** 2021-08-12

**Authors:** Hayrunnisa Bolay, Ömer Karadas, Bilgin Oztürk, Riza Sonkaya, Bahar Tasdelen, Tuba D. S. Bulut, Özlem Gülbahar, Aynur Özge, Betül Baykan

**Affiliations:** 1 Department of Neurology and Algology, Neuropsychiatry Center, Neuroscience and Neurotechnology Center (NÖROM), Gazi University Hospital, Medical Faculty, Besevler, 06510 Ankara, Turkey; 2 Neurology Department, University of Health Science, Gülhane School of Medicine, Ankara, Turkey; 3 Department of Biostatistics and Medical Informatics, Mersin University, Medical Faculty, Mersin, Turkey; 4 Department of Medical Biochemistry, Gazi University, Medical Faculty, Ankara, Turkey; 5 Department of Neurology and Algology, Mersin University, Medical Faculty, Mersin, Turkey; 6 Istanbul Faculty of Medicine, Department of Neurology, Headache Center, Istanbul University, Istanbul, Turkey

**Keywords:** COVID-19, Headache, Inflammation, NLRP3, HMGB1, ACE2, Angiotensin II, IL-6, IL-10, CGRP

## Abstract

**Background and aim:**

Pathogenesis of COVID-19 -related headache is unknown, though the induction of the trigeminal neurons through inflammation is proposed. We aimed to investigate key systemic circulating inflammatory molecules and their clinical relations in COVID-19 patients with headache.

**Methods:**

This cross-sectional study enrolled 88 COVID-19 patients, hospitalized on a regular ward during the second wave of the pandemic. Clinical characteristics of COVID-19 patients were recorded, and laboratory tests were studied.

**Results:**

The mean ages of 48 COVID-19 patients with headache (47.71 ± 10.8) and 40 COVID-19 patients without headache (45.70 ± 12.72) were comparable. COVID-19 patients suffered from headache had significantly higher serum levels of HMGB1, NLRP3, ACE2, and IL-6 than COVID-19 patients without headache, whereas CGRP and IL-10 levels were similar in the groups. Angiotensin II level was significantly decreased in the headache group. COVID-19 patients with headache showed an increased frequency of pulmonary involvement and increased D- dimer levels. Furthermore, COVID-19 was more frequently associated with weight loss, nausea, and diarrhea in patients with headache. Serum NLRP3 levels were correlated with headache duration and hospital stay, while headache response to paracetamol was negatively correlated with HMGB1 and positively associated with IL-10 levels.

**Conclusion:**

Stronger inflammatory response is associated with headache in hospitalized COVID-19 patients with moderate disease severity. Increased levels of the circulating inflammatory and/or nociceptive molecules like HMGB1, NLRP3, and IL-6 may play a role in the potential induction of the trigeminal system and manifestation of headache secondary to SARS-CoV-2 infection.

## Introduction

COVID-19 is characterized by robust systemic inflammation and the release of inflammatory molecules and pro-inflammatory cytokines [1, 2]. Headache is one of the presenting and frequent symptoms of COVID-19 [3–7]. It is intriguing to note that headache is not seen in all COVID-19 patients or is not only prevalent in those primary headache sufferers, such as migraine. Headache frequency is reported to be 12% in a meta-analysis [3] and 29% in hospitalized COVID 19 patients with moderate disease severity [5]. New-onset, unrelenting, moderate to severe, frontally located, pulsating, or pressing headaches, associated with the anosmia/ageusia and gastrointestinal complaints, especially when seen in men, are more frequently associated with COVID-19 [4–8].

The probable pathophysiology of the headache secondary to COVID-19 is suggested by us to involve inflammation, vasculopathy, or direct involvement of the trigeminal nerve in the upper respiratory system [2, 9]. In a prospective clinical study, we demonstrated that COVID-19 headache was associated with inflammation and the headache phenotypes were determined by several factors including elevated circulating IL-6 levels [5]. Renin angiotensin system plays a central role in COVID-19 as SARS-CoV2 infects host cells by binding to angiotensin converting enzyme 2 (ACE2), which metabolizes angiotensin II (Ang II). NLRP3 (Nod-like receptor pyrin domain-containing 3) inflammasome, a critical part of the innate immune defense system can be activated by SARS-CoV-2 and/or Ang II receptor 1 signal transduction pathway [9, 10]. It is hypothesized that dysregulated angiotensin system and NLRP3 inflammasome play a key role in trigeminal induction and headache symptom in COVID-19 [2, 9].

The host immune response depends either on the detection of pathogen-associated molecular patterns (PAMP) or damage signals generated during infections and/or tissue injury. Damage-associated molecular pattern molecules (DAMPs) include high mobility group box-1 (HMGB1) which is an intranuclear protein, present in most eukaryotic cells. HMGB1 participates in regulating critical genomic events as a DNA binding protein, though it can also be transported to the extracellular environment. Once HMGB1 is present in the extracellular space, it acts as a potent proinflammatory mediator [10]. Extracellular HMGB1 binds several receptors, including receptor for advanced glycation end-products (RAGE), toll-like receptors (TLR) receptors. HMGB1 mediated neuroinflammation has been implicated in the pathogenesis of diverse disorders including cerebral injury, persistent pain, and trigeminal neuropathy [10, 11]. Recent studies have also highlighted that HMGB1 is one of the important host factors for COVID-19 pathogenesis [10]. Regarding the disease severity of COVID-19, a correlation with increased inflammatory responses is well-established and HMGB1 is a critical extracellular mediator in these inflammatory processes.

The potential nociceptive role of the elevated circulating pro-inflammatory cytokines because of COVID-19 could play a role in the emergence of headache. The present study aimed to investigate whether serum levels of NLRP3, HMGB1, IL-6, IL-10, angiotensin II and ACE2 were different in COVID-19 patients with headache. We also aimed to determine the relation of the above-mentioned inflammatory mediators and clinical features of headache in patients diagnosed with COVID-19.

## Methods

### Subjects

COVID-19 patients (*n* = 107) hospitalized with moderate symptoms in the routine COVID-19 ward were consecutively recruited during the second wave of the pandemic in January and February 2021 for this cross-sectional study.

Inclusion criteria were COVID-19 diagnosis according to WHO interim guidance, polymerase chain reaction (PCR) positivity for SARS-CoV2, adults aged between 18 and 70 years, without any history of malignancy, severe metabolic syndrome, uncontrolled hypertension, uncontrolled diabetes, chronic liver failure, chronic respiratory diseases such as chronic obstructive pulmonary disease (COPD) or interstitial pulmonary disease, chronic renal failure, decompensated congestive heart failure, current alcohol/drug abuse, and major psychiatric disorders. The presence of a clearly reported headache was required for inclusion in the headache group. Exclusion criteria were patients transferred to ICU, headache attributed to other secondary causes, patients who had severe hearing or speech impairment to interfere with communication and patients who refused further laboratory investigations. COVID-19 patients with mild- moderate headaches, displaying VAS ≤7 (visual analog scale) were excluded, to create reliably differentiating groups with versus without headache. Based on a previous report [5]. Consequently, among 107 patients diagnosed with COVID-19 illness 88 patients were enrolled in the study. Patients, who required mechanical ventilator or ICU stay within the first 10 days of hospitalization (*n* = 3), those diagnosed with other secondary headache etiologies (n = 3), and finally patients with lower VAS ≤7 scores (*n* = 12) were excluded from the study.

The study was performed in accordance with the principles of the Helsinki Declaration and approved by the Local Ethical Committee and Ministry of Health Ethical Committee (Protocol No: 2021–18 and 2021-09 T07–52-03 respectively). Written informed consent was obtained from all patients about the recruitment for scientific purposes. All patients with COVID-19 were diagnosed and managed with the same treatment protocol, approved by the Ministry of Health, consistent with WHO Guidelines [12].

Neurologists and headache specialists examined all COVID-19 patients and questioned them for the absence/presence of headache and past headache history. Headache intensity was determined by VAS (VAS > 7: severe intensity). Other symptoms like anosmia, ageusia, nausea, weight loss and loss of appetite were recorded.

Nasopharyngeal and nasal swab specimens were studied for SARS-CoV-2 real-time reverse-transcription polymerase chain reaction (rRT-PCR) test. Thorax CT and routine laboratory tests (complete blood cell count, blood chemical analysis) were investigated in a standardized manner in all patients. D-dimer test has been performed by using a fluorescence immunoassay for the quantitative determination of cross-linked fibrin degradation products containing D-dimer in EDTA anticoagulated plasma specimens (reference values < 0.50 mcg/mL).

To study inflammatory molecules, a peripheral blood sample was taken from the patients at hospital admission. Serum samples were stored at − 80 degrees until analyzed. Serum NLRP-3, IL-6, IL-10, HMGB-1, ACE2, Ang-II and CGRP levels were measured by ELISA (Enzyme-Linked Immuno Sorbent Assay) method using commercial kits according to the manufacturer’s instructions. Human ELISA kits were obtained from Bioassay Technology Laboratory Shanghai Korain Biotech Co. (NLRP-3), Elabscience Biotechnology Inc. (IL-6, IL-10, HMGB-1, ACE2, Ang-II) and ELK Biotechnology (CGRP). Intra-assay and inter-assay CV values ​​of methods were < 10%. Researchers conducted laboratory tests and data analyst were blinded to the allocation of the patients.

### Statistical analysis

Continuous data were summarized as mean ± standard deviation and median (Q1-Q3) values and categorical data were described with count (%) values. Shapiro Wilk’s test was used to check normality. The comparisons of COVID-19 patients with and without headache were made using independent samples t test (mean age, HMGB1) or Mann Whitney U test (median NLRP3, IL-6, IL-10, angiotensin II, ACE-2, D-dimer). The relationship between categorical variables was evaluated by means of chi-square test and Fisher’s exact test. To control familywise error rate in these comparisons about main hypothesis of study, Bonferroni adjustment was applied. In addition, Receiver operating characteristics (ROC) curves were used to obtain cut-off values associated with COVID-19 headache. It is simply based on the density estimation of variables that are observed for the case concerned. Pairwise comparisons of area under ROC curves (AUC) of cytokines were performed using Delong’s method. Multiple logistic regression was used to evaluate the combined performance of ACE2, HMGB1, IL-6 and NLRP3 and AUC was obtained. This is also used to interpret the prediction power of the model to classify positive and negative cases [13]. In addition, we acquired percent correct classification values (also called Sensitivity and Specificity).

In the study, STATISTICA 13.0 (TIBCO Software Inc.) and MedCalc 19.8 (free trial version) software was used, and statistical significance level was set at *p* < 0.05 [13, 14].

In addition, for modeling and visualization of associations between multiple variables, Network analysis, a multivariate graphical technique, was used. The networks analysis, a relatively new and promising method for modeling interrelationships between large numbers of variables were performed by using the JASP software version 0.14 [15]. Networks are composed of nodes and lines (edges). In this study, we used correlations as weights because the correlation network provides a lot of information about the data, all edges are shown, not just the significant ones [15]. The thicker the connection between nodes, the stronger correlation between variables [16]. Moreover, the color of edges represents either positivity (blue) or negativity (red) of correlations.

## Results

### Demographic features, clinical and laboratory characteristics

Demographic features, clinical characteristics, and laboratory results of the two groups with and without headache were given in Table 1. Male dominance was notable in COVID-19 patients with headache; age distribution was similar in both groups. All patients received the same standard treatment protocol including low molecular weight heparin according to the regulations of Ministry of Health. None of the included COVID-19 patients suffered from hypoxia or required ventilation or had any serious complication including coagulopathy problem during their stay in the hospital.

**Table 1:** Demographic features, clinical characteristics, and laboratory results of the two groups with and without headache

	COVID-19 Without Headache *n* = 40	COVID-19 With Headache *n* = 48	*P* value
*Mean Age (years)*	45.70 ± 12.72	47.71 ± 10.78	0.425
*Women/ Men*	19/21	16/32	0.176
*Primary headache*	3	11	0.077
*Anosmia*	1	7	0.066
*Ageusia*	1	5	0.214
*Nausea*	3	23	< 0.001
*Loss of appetite*	7	29	< 0.001
*Weight loss*	4	25	< 0.001
*Diarrhea*	7	15	0.216
*Pulmonary involvement*	13	31	0.003
*D-dimer (mg/mL)*	1.22 ± 1.18	8.42 ± 7.07	< 0.001
*Hospitalization (days)*	6.08 ± 1.37	8.38 ± 2.42	< 0.001

### Headache characteristics

The presence of severe headache was significantly associated with nausea, loss of appetite, and loss of weight (*p* < 0.001) compared to COVID-19 patients without headache. The frequency of history of primary headache was not statistically different between the two groups. Pulmonary involvement was detected in 44 patients with COVID-19 and among them, 31 had headache with VAS > 7. Hospitalization was about 2 days longer in COVID-19 patients with headache compared to non-headache sufferers (Table 1). Headache was bilateral, severe, pulsating/pressing quality and unrelenting in COVID-19 patients that was consistent with our previous reports in other series [4, 5, 8]. COVID-19 related headache was unresponsive to paracetamol in 75% of the patients and its duration was 5.1 ± 1.2 days (median:5, range 4–8 days).

### Other clinical associations

COVID-19 patients with severe headache showed a higher ratio of pulmonary involvement diagnosed by thorax CT imaging compared to COVID-19 patients without headache (65% vs 33%, *p* < 0.001) (Table 1). Furthermore, hospitalization days were less in COVID-19 patients without pulmonary infiltration compared to that of with pulmonary involvement (7.8 ± 2.4 vs 6.8 ± 2.08 *p* = 0.03). In terms of routine laboratory parameters, D-dimer levels were significantly higher in COVID-19 patients with severe headache (median: 6.20; 4.80–8.80) compared to those COVID-19 patients without headache (median: 0.75;0.20–1.95, *p* < 0.001). COVID-19 patients with pulmonary involvement (*n* = 44) exhibited more frequent loss of appetite (*n* = 26 vs *n* = 10, *p* = 0.001), loss of weight (*n* = 21 vs *n* = 8, *p* = 0.006) and nausea (*n* = 20 vs *n* = 6, *p* = 0.002). Symptoms of anosmia, ageusia, and diarrhea were not different in COVID-19 patients with pulmonary involvement compared to COVID-19 patients without pulmonary involvement.

### Levels of inflammatory and nociceptive molecules and their clinical relations

**Fig. 1:**
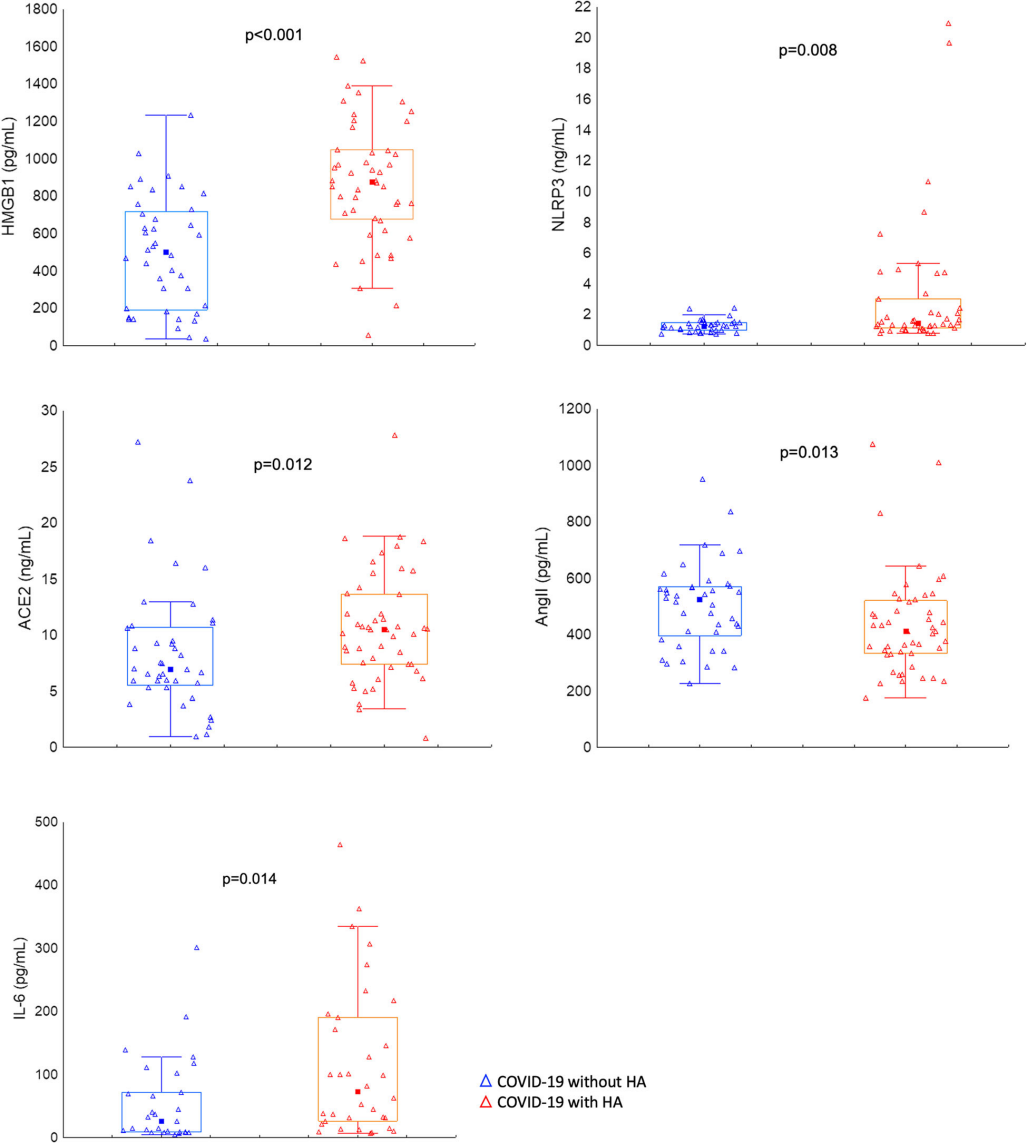
Serum levels of inflammatory molecules at hospital admission in COVID-19 patients. HMGB1, NLRP3, ACE2, IL-6 levels were significantly increased in COVID-19 patients with headache (*n* = 48) compared to COVID-19 patients without headache (*n* = 40). Angiotensin II level was significantly decreased in COVID-19 patients with headache

**Fig. 2:**
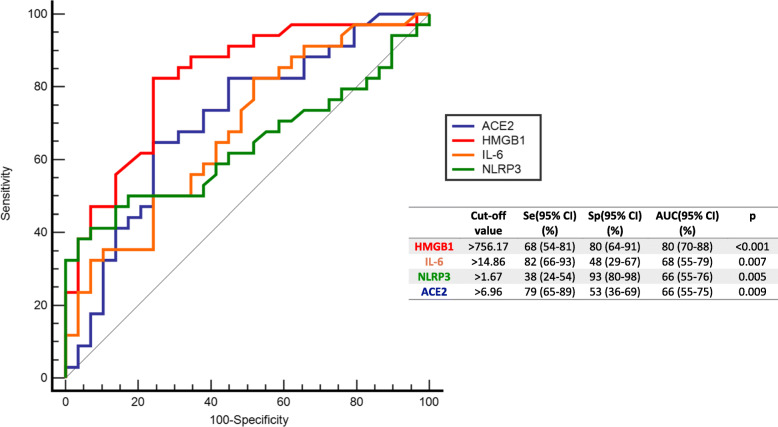
Independent ROC curves illustrated the differentiating abilities of HMGB1, IL-6, NLRP3 and ACE2 (*n* = 88). Both sensitivity (Se) and specificity (Sp) values showed that classification performance of HMGB1 was better than the others. On the other hand, IL-6 and ACE2 were found to be successful in classifying COVID-19 patients with headache, while NLRP3 were found to be more successful in classifying COVID-19 patients without headache. Combined performance of these inflammatory molecules was found to be highly successful (Area under curve AUC = 0.92, Se = 0.83, Sp = 0.88)

Compared to COVID − 19 patients without headache, serum levels of HMGB1, NLRP3, IL-6, angiotensin II, and ACE2 were significantly higher in COVID-19 patients with severe headache (Figs. 1 and 2). Serum CGRP levels were not statistically significant between COVID-19 without headache (median:40.23 (21.13–63.42) and with headache (median: 36.37 (25.02–86.59, *p* = 0.456). Serum IL-10 levels were not different between the two groups (median 13.01 (9.54–18.79) vs (median: 13.30 (10.98–20.24), *p* = 0.157).

**Fig. 3:**
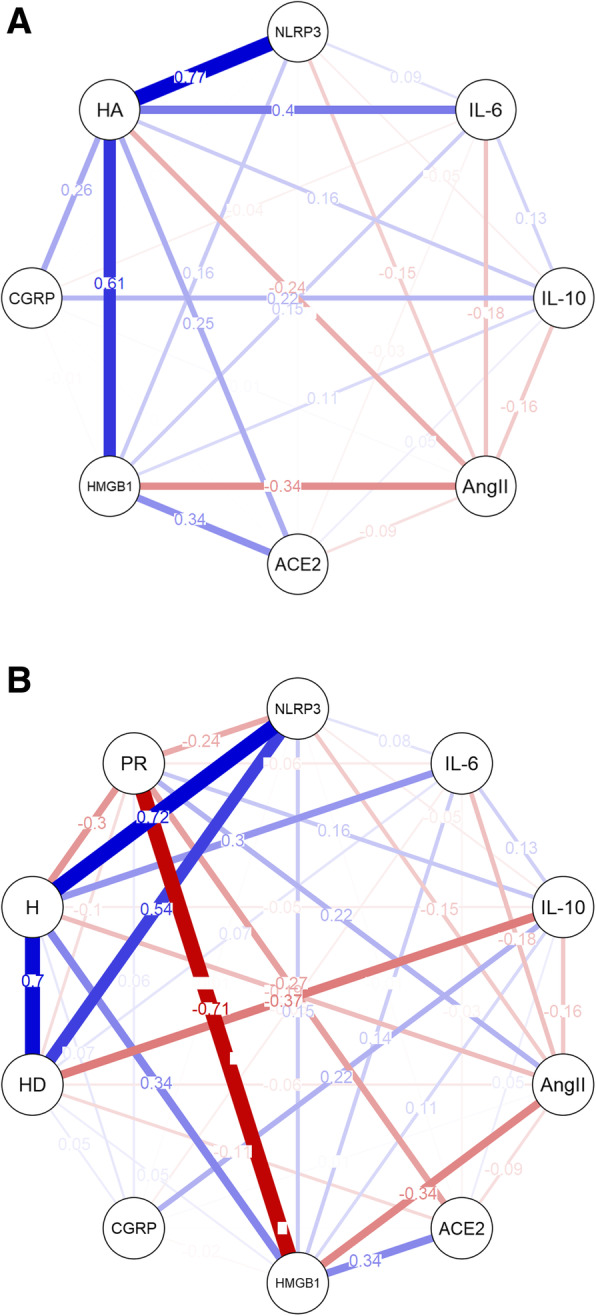
Network analysis models of the interrelationships among inflammatory molecules, headache (HA), headache duration (HD), hospitalization (H) and paracetamol response (PR) in COVID-19 patients with headache (*n* = 48). Headache was positively correlated with serum levels of NLRP3, HMGB1, IL-6 and ACE2 and these relationships were greater than the others (**A**). Serum HMGB1 level was negatively correlated with paracetamol response (w = −0.71) (B). Hospitalization (w = 0.72) and headache duration (w = 0.54) were positively correlated with NLRP3 level (**B**). Line thickness denotes weight of the correlation, blue and red colors represent positive and negative correlations, respectively

COVID-19 patients with headache and pulmonary infiltration (*n* = 31) had significantly higher serum levels of HMGB1, IL-6, D-dimer (Table 2). NLRP3 was correlated with headache duration (w = 0.69) and hospital stay (w = 0.63) in COVID-19 patients with headache (Fig. 3). Response of headache to paracetamol 1000 mg was significantly lower in patients with pulmonary involvement (13% vs 47%, *p* = 0.008). In COVID-19 headache patients who are unresponsive to paracetamol, HMGB1 level was significantly elevated (989.3 ± 263.5 vs 514.7 ± 248.9, *p* < 0.001) whereas IL-10 level (16.1 ± 11.9 vs 19.4 ± 5.6 *p* = 0.017) was decreased.

**Table 2:** Comparison of the inflammatory molecule levels in COVID-19 patients with headache regarding pulmonary involvement

	COVID-19 Patients with Headache (*n* = 48)				
	Pulmonary Infiltration (***n*** = 31) 65%		Without Pulmonary Infiltration (***n*** = 17) 35%		
	**Mean ± SD**	**Median** **(Q1-Q3)**	**Mean ± SD**	**Median** **(Q1-Q3)**	***P*value**
NLRP3 (ng/mL)	3.03 ± 3.92	1.43 (1.15–3.32)	3.18 ± 4.91	1.53 (1.11–2.70)	0.982
IL-6 (pg/mL)	141.63 ± 126.2	100.09 (26.04–217.05)	34.13 ± 18.83	31.84 (20.70–48.39)	0.036
IL-10 (pg/mL)	18.12 ± 12.46	13.58 (10.98–20.24)	14.96 ± 6.37	12.72 (10.40–19.66)	0.283
Ang II (pg/mL)	416.22 ± 188.78	366.45 (267.33–524.59)	471.62 ± 165.93	443.07 (401.79–514.73)	0.111
ACE2 (ng/mL)	11.65 ± 5.40	10.71 (7.36–15.72)	9.02 ± 3.63	8.93 (7.39–10.60)	0.073
HMGB1 (pg/mL)	960.17 ± 318.71	964.87 (724.42–1205.49)	707.48 ± 294.84	759.38 (485.12–923.55)	0.012
D-dimer (μg/mL)	10.26 ± 8.00	7.20 (5.50–13.90)	5.05 ± 2.82	4.90 (2.90–6.80)	0.011
CGRP (pg/mL)	86.13 ± 139.05	34.72 (25.02–67.22)	208.48 ± 290.35	55.46 (20.92–287.63)	0,321

Q1: Lower Quartile; Q3: Upper Quartile

### Receiver operating characteristics curves

ROC for inflammatory markers indicated the significant role of HMGB1, NLRP3, IL-6 and ACE2 in discriminating the presence of headache in patients with COVID-19 (Fig. 2). Both sensitivity (Se: 0.68; 95% CI: 0.54–0.81) and specificity (Sp: 0.80; 95% CI: 0.64–0.91) showed that independent classification performance of HMGB1 was better than the others (AUCHMGB1: 0.80 95% CI: 0.70–0.88; AUCIL-6: 0.68 95% CI: 0.55–0.79; AUCNLRP3: 0.66 95% CI: 0.55–0.76; AUCAACE2: 0.66 95% CI: 0.55–0.75;). On the other hand, IL-6 and ACE2 were found to be successful in classifying COVID-19 patients with headache (SeIL-6: 0.82; SpIL-6:0.48) (SeACE2: 0.79; SpACE2: 0.53), while NLRP3 was successful in classifying COVID-19 patients without headache (SeNLRP3: 0.38; SpNLRP3:0.93). We observed that independent classification performances of inflammatory markers were unbalanced. Hence, we considered multivariate structure between these markers and obtained combined performance of them. Combined performance of these inflammatory molecules was found to be highly successful for discriminating headache in COVID-19 (Area under curve AUC = 0.92, Se = 0.83, Sp = 0.88). It was highly successful (Area under curve AUC = 0.92, Se = 0.83, Sp = 0.88).

### Network analysis

Network plot analysis illustrated the positive and negative relationships among inflammatory molecules and COVID-19 headache (HA) (Fig. 3A), headache duration (HD), hospital stay (H) and paracetamol response (PR) (Fig. 3B). There was a moderately high positive association between NLRP3 and HA (w = 0.77), H (w = 0.72) and HD (w = 0.54). HMGB1 was positively associated with HA (w = 0.61) and was negatively associated with PR (w = − 0.71). According to Fig. 3A and Fig. 3B, CGRP was not associated with any of the markers except IL-10 (w = 0.11–0.14).

## Discussion

This study supplied evidence that the presence of severe headache in COVID-19 was associated with elevated inflammatory and/or nociceptive molecules like HMGB1, NLRP3, IL-6, and ACE2 compared to the non-headache COVID-19 sufferers, while anti-inflammatory cytokine levels of IL-10 did not show any difference at hospital admission between the two groups. Moreover, NLRP3 and HMGB1 were correlated with headache duration and paracetamol unresponsiveness respectively. A robust inflammatory response was detected particularly in COVID-19 patients with severe headache. In support, these patients were also associated with increased frequency of pulmonary involvement but not needing any ICU management.

Angiotensin-converting enzyme 2 (ACE2) has gained importance in COVID-19 pathogenesis because membrane-bound ACE2 is the host receptor for SARS-CoV-2 [17]. ACE2 is widely expressed in the human body, including epithelial cells in nasal and oral mucosa, pneumocytes in the respiratory system, besides the vascular endothelial cells and smooth muscle cells [18]. Soluble ACE2 on the other side could reflect the cleavage of membrane-bound ACE2 during the SARS-CoV-2 entry and lysis of ACE2-expressing cells. High levels of plasma ACE2 is proposed to indicate poor outcome in critically ill patients [19, 20]. The moderate disease course was different in our patients, in whom elevated ACE2 levels were seen in association with good outcomes without any ARDS, serious complication or mortality. Increased circulating ACE2 level is even suggested to denote endogenous protection against SARS-CoV-2 by blocking its cell entry [21, 22].

Angiotensin II levels detected in our study merit particular emphasis. Unless cleaved by ACE2 enzyme, angiotensin II peptide mediates various functions including regulation of blood pressure, vascular tonus, electrolyte balance, and induce inflammation, produce reactive oxygen species, and activate NLRP3 inflammasome [2, 9, 17, 23, 24]. The internalization of membrane bound ACE2 by the virus binding would result in the unbalanced activity of angiotensin II, yielding NLRP3 inflammasome activation and release of pro-inflammatory cytokines subsequently recruiting inflammatory cells [2, 9, 18, 23]. Angiotensin II is also involved in pain signaling and induce nociceptive behavior [25]. Besides, angiotensin II receptor inhibitors are implicated as being effective in migraine and neuropathic pain treatments [9, 26]. Consequently, elevated circulating angiotensin II levels in COVID-19 patients were presumed to contribute to trigeminal nociception and headache [2, 9]. However, our study failed to show higher levels of angiotensin II in serum samples of COVID − 19 patients with headache. On the contrary, angiotensin II levels in the systemic circulation were significantly decreased in parallel to elevated ACE2 levels in COVID-19 patients with headache. It is likely that increased circulating ACE2 may break down angiotensin II and be responsible for the lower levels of serum angiotensin II at the point of our measurements in COVID-19 patients. There are conflicting reports on the levels of Ang II peptide in COVID-19 patients. Higher plasma Ang II level was reported in COVID-19 patients with severe symptoms compared to mild cases [27], whereas other investigators detected no alterations in the serum levels of renin–angiotensin–aldosterone system peptides in COVID-19 cases [28]. On the other hand, a significantly lower serum Ang II level was reported in patients hospitalized with COVID 19 [29], which is consistent with our findings. Taken all together, our results may suggest that decreased circulating angiotensin II is implausible to be responsible for the trigeminal nociception in COVID-19 patients.

Likewise, a potential role of serum CGRP in trigeminal induction is less likely in our study, as systemic circulating CGRP levels were comparable in COVID-19 patients with or without headache. The role of CGRP in COVID-19 is largely unknown but Ochoa-Callejero and colleagues reported significantly decreased serum CGRP levels in COVID-19 patients [30]. Accordingly, we can assume that headache symptom in our COVID-19 patients cannot be attributed to circulating CGRP. It is noteworthy, however, that the level of circulating CGRP does not necessarily reflect CGRP release from trigeminal neurons.

Elevated HMGB1 level is significantly associated with the emergence of severe headache and correlated with paracetamol unresponsiveness in our patients with COVID-19 headache. HMGB1 as a prototypical DAMP represents a critical marker of intense inflammation and studies have shown that serum HMGB1 is increased in COVID-19 cases and positively correlated with disease severity. Moreover, serum HMGB1 also decreases when patients improved [31, 32] and regulates autophagy, which is related to SARS-CoV-2 entry. HMGB1 mediates nociceptive action through pattern recognition receptors, such as RAGE, TLR4 and TLR2 [11, 33, 34]. Dorsal root ganglia sensory neurons and trigeminal neurons were shown to express RAGE and TLR4. In primary afferent neurons, mostly responsive to capsaicin, HMGB1 was shown to increase calcium mobilization and neuronal excitability dependent on RAGE [35, 36]. Also, immune cells and glial cells within the close vicinity of nociceptors express these receptors and neuron-glia signaling is increased by HMGB1 [33–36]. HMGB1 induces mechanical hypersensitivity and the blockade of TLRs, and RAGE has been shown to reduce hypersensitivity and pain behavior in neuropathic pain models. Treatment of the trigeminal nerve with anti-HMGB1 neutralizing antibody prevented pain behavior and blocked macrophage and microglia activation [37]. Therefore, HMGB1 could be a key player in the development of severe and long-lasting COVID-19 headache. Our results indicated that it will be important to study the potential role of HMGB1 in COVID-19-related headache in related models to gain more insight into the headache mechanisms triggered by external sources, specifically in COVID-19.

We observed that the serum level of NLRP3 inflammasome was significantly increased in COVID-19 patients with headache. Activation of inflammasome is a complex process induced by diverse stimuli including SARS-CoV2, HMGB1 or cytokines and release pro-inflammatory cytokines such as IL-1β, IL18 to amplify local and systemic inflammatory response [9, 10, 23, 24, 38]. Neurogenic inflammation, neuropeptides and NLRP3 inflammasome also plays a complex role in various experimental models of migraine headache [39, 40]. Recently, the dysregulated NLRP3 inflammasome activation that releases nociceptive cytokines is proposed in COVID-19 headache pathophysiology [9]. Network plot analysis showed that NLRP3 was associated with the presence of headache in COVID-19 and correlated with both the duration of headache and the duration of hospital stay in our study. In glyceryl trinitrate-induced experimental headache studies, activation of NFκβ, IL1β, IL-6 and NLRP3 inflammasome are shown within perivascular macrophages in the dura mater and in microglial cells nearby trigeminal sensory neurons in the brainstem [41–43]. However, the exact role of circulating NLRP3 levels in inducing trigeminal nociception needs to be determined by further studies.

Pro-inflammatory cytokine IL-6 plays a key role in both peripheral and central neuroinflammation that was shown in several pathological pain models [44]. In the trigeminal system, IL-6 can activate perivascular trigeminal nociceptors in the dura mater and induce headache in behavioral experimental studies [45, 46]. IL6 acts through IL-6 receptor and IL-6 signal-transducer glycoprotein 130 (gp130) that are expressed in sensory nociceptive neurons and activates the intracellular ERK pathway [47]. Additionally, proinflammatory IL-6 cytokine can provoke CGRP release particularly under heat conditions [48]. Therefore, higher circulating levels of IL-6 in patients with more intense headache could point out a potential trigger role of IL-6 in COVID-19 headache.

Pulmonary involvement was detected by thorax CT in half of all patients and 65% of the COVID-19 headache sufferers. Intriguingly, D-dimer levels were significantly higher in patients with headache compared to non-headache sufferers. Increased serum D-dimer has been considered to reflect coagulopathy and poor prognosis in severe COVID-19 patients [49], but we observed no thrombotic event or mortality in our study group. In COVID-19 patients with moderate disease severity, higher levels of IL-6 and D-dimer with increased frequency of pulmonary infiltration were associated with severe headache [5]. As proposed by Hunt & Levi, D-dimer levels could represent the degree of lung inflammation, like other acute-phase proteins in COVID-19 [50]. The latter would provide a rational explanation for the correlation of D-dimer elevation and pulmonary infiltration in our COVID-19 patients with headache. Moreover, the presence of headache in hospitalized COVID-19 patients is stated as an independent predictor of lower risk of mortality [51]. Our results are different, as COVID-19 patients with hypoxia and who required ventilation in the ICU were not included in our study group. Yet, 35% of the patients without any pulmonary infiltration (Table 2) manifested severe headache intensity and elevation of IL-6, HMGB1. It seems that circulating inflammatory cytokines could also be associated with headache without pulmonary inflammation detected by CT.

We think that the headache correlated with the presence of pulmonary infiltration cannot merely be attributed to hypoxia as severe hypoxemia and/or hypercapnia were not identified in any of our patients. Rather, we propose pro-inflammatory mediators and cytokines released extremely during pulmonary infiltration could play a key role in the development of headache in the COVID-19 course. A recent study revealed that IL-6 levels were positively correlated with VAS scores in COVID-19 patients with headache [5]. Also, ROC curve cut-off values were reported to indicate a moderate increase of IL-6 for defining COVID-19 headache [5]. Both IL-6 and IL-10 are reported to be predictors of COVID-19 severity [52]. The discordance of these cytokines, we found in COVID-19 patients with versus without headache, deserves special attention. IL-10, a typical anti-inflammatory cytokine did not show any difference in relation to the presence of headache, whereas IL-6, a major pro-inflammatory cytokine showed significantly higher levels in COVID-19 patients with headache, intriguingly. Even though IL-6 and IL-10 were noted as predictors for COVID-19 deterioration, all of our patients were followed up in the hospital but not in ICU. The similarity of the IL-10 levels could be due to the early sampling at the hospital admission, its course would be different during the COVID-19 process.

Taken all the data together, we found significant elevations of circulating HMGB1, NLRP3 and IL6 levels in COVID-19 patients with headache compared to COVID-19 patients without headache. The possible molecular mechanisms of trigeminal activation by these circulating proinflammatory and/or nociceptive molecules are striking. Our data suggests that HMGB1 as a potent proinflammatory molecule with nociceptive properties may be a central player in proinflammatory reactions during SARS-Co-V2 infection and induction of trigeminal neurons.

In terms of deeper look, extracellular HMGB1 is secreted from activated macrophages, monocytes natural killer cells and dendritic cells in response to pathogen invasion such as SARS-CoV2 [10, 31]. Neurons, astrocytes, microglia, and endothelial cells are also able to actively secrete HMGB1 after exposure to cytokines, interferons, and NO [40, 47]. Furthermore, cell injury and necrotic process can contribute to extracellular HMGB1 increase. HMGB1 binding to RAGE, TLR4 or TRL2 is critical in amplifying inflammatory processes, and subsequent production of pro-inflammatory cytokines in different cell types. RAGE activates diverse intracellular signal transduction pathways including the p38 MAPK, the ERK-1/2 kinases, rho-GTPases, the NF-κB pathway and STAT3 [53]. HMGB1/RAGE/TRL axis increases the expression of NLRP3 inflammasome components including NLRP3, ASC and caspase-1 and consequent IL-1β production [54–56]. The NLRP3 activation by HMGB1 and IL-1β production was shown to be dependent on NF-κB pathway [55] that release proinflammatory cytokines, including IL-6, and IL-1β, and play major role in neuroinflammation.

Recently a novel mechanism was shown that inflammasome proteins can be transported within extracellular vesicles from the brain to the lungs and induce acute lung injury [57]. Therefore, we suggest that HMGB1, released mainly from respiratory infection in COVID-19 may convey the damage signal to the trigeminal neurons in the ganglia and trigeminal perivascular nociceptors within the dura mater and activate the trigeminal system through pattern recognizing receptors. Moreover, proinflammatory cytokines and inflammasome proteins in the extracellular space may act on the sensory neurons in the trigeminal ganglia, perivascular nociceptors in the dura mater. The trigeminal activation drives corresponding headache pathways and releases inflammatory/ nociceptive molecules that amplifies inflammatory response and reciprocal signaling between cytokine-generating cells.

Our clinical study has some constraints, as the study was conducted in hospitalized patients in the general ward, without including patients in the ICU and non-hospitalized mild symptomatic patients. In future studies, other groups with COVID-19 headaches like those with significant comorbidities, those transferred to the ICU, and subjects with mild/moderate (VAS ≤7) headaches should be investigated prospectively to see the clinical relations and changes of the cytokines over time. Another potential limitation was that our results may indicate more severe involvement in cases with headache, reflecting a potential recruitment bias. We were able to study a limited number of inflammatory molecules at hospital admission only and follow-up data providing their temporal progression during COVID-19 headache are lacking in the current work.

This pioneering study on headache in COVID-19 has several implications. So far, it is the first clinical study investigating the role of circulating inflammatory and nociceptive molecules such as HMGB1, NLRP3 inflammasome, IL-6, IL-10, angiotensin II, and ACE2 in COVID-19 headache. Elevated HMGB1, NLRP3 and IL-6 levels could be discriminative markers for COVID-19 headache and may have implications for prevention and also for other secondary headaches related to other systemic viral infections. No significant association of serum CGRP level with COVID-19 related headache is notable and raises the importance of HMGB1, NLRP3, IL-6 in COVID-19 headache pathogenesis. NLRP3 was correlated with headache duration and hospital stay, while paracetamol response was negatively associated with HMGB1 levels and positively correlated with IL-10 levels. Moreover, the current study conducted during a different wave of the pandemic confirmed the previous report that higher IL-6 levels and more frequent pulmonary infiltration were detected in COVID-19 patients with headache.

## Conclusion

We conclude that significant elevation of HMGB1, NLRP3, and IL-6 levels could induce trigeminal activation leading to headache during the SARS-COV-2 infection. This significant participation of circulating inflammatory mediators in trigeminal nociception does not necessarily exclude the possible involvement of trigeminal nerve activation, by SARS-CoV-2 directly or secondary to vasculopathy.

## Article highlights

- COVID-19 patients with headache had stronger inflammatory response and significantly elevated serum levels of HMGB1, NLRP3, ACE2, and IL-6 than COVID-19 patients without headache.
- Serum NLRP3 levels were correlated with headache duration and hospital stay, while headache response to paracetamol was negatively correlated with HMGB1 levels.
- Elevated levels of the circulating inflammatory and/or nociceptive molecules like HMGB1, NLRP3, and IL-6 may play a role in the potential induction of the trigeminal neurons and manifestation of headache secondary to SARS-CoV-2 infection.

## Data Availability

The datasets generated during and/or analyzed during the current study are not publicly available as the signed informed consent form by all patients did not include a provision stating that individual raw data can be made publicly accessible but are available from the corresponding author on reasonable request.
